# Supplementary material for: Clinical and cost evaluation of intensive support team (IST) models for adults with intellectual disabilities who display challenging behaviour: a comparative cohort study protocol
Source: BMJ Open. 2021 Mar 30;11(3):e043358. doi: 10.1136/bmjopen-2020-043358 (PMC8011791; doi:10.1136/bmjopen-2020-043358)
Supplement: Author's manuscript [file bmjopen-2020-043358.draft_revisions.pdf]

BMJ Open is committed to open peer review. As part of this commitment we make the peer review history of every article we publish publicly available.

When an article is published we post the peer reviewers' comments and the authors' responses online. We also post the versions of the paper that were used during peer review. These are the versions that the peer review comments apply to.

The versions of the paper that follow are the versions that were submitted during the peer review process. They are not the versions of record or the final published versions. They should not be cited or distributed as the published version of this manuscript.

BMJ Open is an open access journal and the full, final, typeset and author-corrected version of record of the manuscript is available on our site with no access controls, subscription charges or pay-per-view fees (http://bmjopen.bmj.com).

If you have any questions on BMJ Open's open peer review process please email info.bmjopen@bmj.com

BMJ Open

Clinical and cost evaluation of intensive support team (IST) models for adults with intellectual disabilities who display challenging behaviour: a comparative cohort study protocol

Journal:	*BMJ Open*
Manuscript ID	bmjopen-2020-043358
Article Type:	Protocol
Date Submitted by the Author:	03-Aug-2020
Complete List of Authors:	HASSIOTIS, ANGELA; University College London, Division of Psychiatry<br>Kouroupa, Nancy; University College London, Division of Psychiatry<br>Jones, Rebecca; University College London, Division of Psychiatry<br>Morant, Nicola; University College London, Division of Psychiatry<br>Courtenay, Ken; Barnet Enfield and Haringey Mental Health NHS Trust<br>Hall, Ian; East London NHS Foundation Trust<br>Crossey, Vicky; NHS Lothian<br>Romeo, Renee; King's College London, Institute of Psychiatry Psychology and Neuroscience<br>Taggart, Laurence; University of Ulster Institute of Nursing and Health Research<br>Langdon, Peter; University of Warwick Centre for Educational Development Appraisal and Research<br>Ratti, Victoria; University College London, Division of Psychiatry<br>Kirchner, Vincent; Camden and Islington NHS Foundation Trust<br>Lloyd-Evans, Brynmor ; UCL, Division of Psychiatry
Keywords:	PSYCHIATRY, Adult psychiatry < PSYCHIATRY, Delirium & cognitive disorders < PSYCHIATRY, Organisation of health services < HEALTH SERVICES ADMINISTRATION & MANAGEMENT

SCHOLARONE™
Manuscripts

BMJ

I, the Submitting Author has the right to grant and does grant on behalf of all authors of the Work (as defined in the below author licence), an exclusive licence and/or a non-exclusive licence for contributions from authors who are: i) UK Crown employees; ii) where BMJ has agreed a CC-BY licence shall apply, and/or iii) in accordance with the terms applicable for US Federal Government officers or employees acting as part of their official duties; on a worldwide, perpetual, irrevocable, royalty-free basis to BMJ Publishing Group Ltd ("BMJ") its licensees and where the relevant Journal is co-owned by BMJ to the co-owners of the Journal, to publish the Work in this journal and any other BMJ products and to exploit all rights, as set out in our licence.

The Submitting Author accepts and understands that any supply made under these terms is made by BMJ to the Submitting Author unless you are acting as an employee on behalf of your employer or a postgraduate student of an affiliated institution which is paying any applicable article publishing charge ("APC") for Open Access articles. Where the Submitting Author wishes to make the Work available on an Open Access basis (and intends to pay the relevant APC), the terms of reuse of such Open Access shall be governed by a Creative Commons licence – details of these licences and which Creative Commons licence will apply to this Work are set out in our licence referred to above.

Other than as permitted in any relevant BMJ Author's Self Archiving Policies, I confirm this Work has not been accepted for publication elsewhere, is not being considered for publication elsewhere and does not duplicate material already published. I confirm all authors consent to publication of this Work and authorise the granting of this licence.

For peer review only

Clinical and cost evaluation of intensive support team (IST) models for adults with intellectual disabilities who display challenging behaviour: a comparative cohort study protocol

Angela Hassiotis[1], Nancy Kouroupa[1], Rebecca Jones[1], Nicola Morant[1], Ken Courtenay[2], Ian Hall[4], Elisabeth Victoria Crossey[3], Renee Romeo[5], Laurence George Taggart[6], Peter E. Langdon[7], Victoria Ratti[1], Vincent Kirchner[8] and Brynmor Lloyd-Evans[1]

Author affiliations

1. Division of Psychiatry, University College London, UK
2. Barnet, Enfield and Haringey Mental Health NHS Trust, London, UK
3. NHS Lothian, UK
4. East London NHS Foundation Trust, UK
5. Institute of Psychiatry Psychology and Neuroscience, King's College London, UK
6. Institute of Nursing and Health Research, Ulster University, UK
7. Centre for Educational Development, Appraisal and Research, University of Warwick, UK and Coventry and Warwickshire Partnership NHS Trust
8. Camden and Islington NHS Foundation Trust, UK

Correspondence to

Prof Angela Hassiotis, UCL Division of Psychiatry, Maple House, 149 Tottenham Court Road, London, W1T 7NF, UK. Email: a.hassiotis@ucl.ac.uk

For peer review only

Abstract

Introduction

Approximately 17% of adults with intellectual disabilities living in the community display behaviours that challenge. Intensive Support Teams (ISTs) have been recommended to provide high quality responsive care aimed at avoiding unnecessary admissions and reducing lengthy inpatient stays in England. We have identified two models of ISTs (model 1: enhanced provision and model 2: independent provision). This study aims to investigate the clinical and cost effectiveness of the two models of ISTs.

Methods and analysis

A cohort of 226 adults with intellectual disability (ID) displaying behaviour that challenges who receive support from ISTs from each model will be recruited and assessed at baseline and 9 months later to compare the clinical and cost effectiveness between models. The primary outcome is reduction in challenging behaviour measured by the Aberrant Behaviour Checklist-Community (ABC-C). The mean difference in change in ABC score between the two IST models will be estimated from a multilevel linear regression model. Secondary outcomes include mental health status, clinical risk, quality of life, health related quality of life, level of functioning and service use. We will undertake a cost effectiveness analysis taking both a health and social care and wider societal perspective. Semi-structured interviews will be conducted with multiple stakeholders (i.e., service users, paid/family carers, IST managers/staff) to investigate the experience of IST care as well as an online survey of referrers to capture their contact with the teams.

Ethics and dissemination

The study was approved by the London–Bromley Research Ethics committee (REC reference: 18/LO/0890). Informed consent will be obtained from the person with intellectual disability, or a family/nominated consultee for those lacking capacity and from his/her caregivers. The findings of the study will be disseminated to academic audiences, professionals, experts by

For peer review only

experience and arm's length bodies and policy makers via publications, seminars, and digital platforms.

Registration number

ClinicalTrials.gov Identifier: NCT03586375

Strengths and limitations of this study

- This study will be the first national evaluation of IST models for adults with intellectual disability.
- Accessing the experience of stakeholders (e.g. IST managers, staff members, family and paid carers and service users) will enable an in depth understanding of how ISTs function and how they respond to people with complex needs.
- This is not a randomised controlled trial so may be subject to biases associated with observational research.

Keywords

Intellectual disability, challenging behaviour, adult, intensive support, positive behaviour

For peer review only

Introduction

People with intellectual (also called learning) disabilities (ID), characterised by cognitive delay and difficulties in adaptive functioning, constitute approximately 1% of the population[1]. Seventeen percent of adults with intellectual disabilities, living in the community present with serious challenging behaviour including aggression, self-injury or other socially inappropriate behaviours[2]. As many as 100,000 children and adults are estimated to be at risk of admission to inpatient care due to the presence of such behaviours if they are not effectively supported in the community[3]. There are ongoing concerns that these individuals are subject to increased rates of hospitalisation, unnecessary long term use of psychotropic medication, poorer health, abuse and exclusion[4].

Intensive Support Teams (ISTs) are specialist teams which have been advocated for many years as the best services to help people with intellectual disabilities and challenging behaviour remain within their local communities. The teams may be staffed by one or more professions (e.g. psychology, nursing, psychiatry). They deliver interventions such as positive behaviour support, support adults with intellectual disabilities living in the community who are in a mental or behavioural crisis, and provide inreach when a person is admitted to a psychiatric inpatient facility. ISTs are recommended to provide high quality proactive and responsive care aimed at avoiding unnecessary admissions or reducing inpatient length of stay and maintaining people in their local community[5-6]. However, there is little evidence to recommend a preferred IST model or way of implementing these aims, and there has not been a comprehensive attempt to describe the clinical and cost effectiveness of ISTs. Such information would be needed by health funders in order to fund services fit for purpose. The National Institute of Health and Care Excellence (NICE) Guideline (NG11)[7] reports that: *"It is widely recognised that locally accessible care settings could be beneficial and could reduce costs but there is no strong empirical evidence to support this"*.

Previous studies describe either demonstration projects following the closure of institutions[8-9] or region wide implementation of stand-alone services[10]. Three small randomised controlled trials (RCTs) with individuals with intellectual disability and challenging behaviour examined: 1) a stand-alone specialist support service delivering Applied Behaviour Analysis in one area in England[11]; 2) a stand-alone team delivering assertive outreach in inner London[12]; and 3) an active case management model[13]. Comparator treatment was usual care in all three studies. The remit of the ISTs differed between studies, with one providing support to people with challenging behaviour whilst the other two provided a replication of the intensive support management from adult mental

For peer review only - http://bmjopen.bmj.com/site/about/guidelines.xhtml

health care. These small studies presented equivocal findings in terms of reduction in challenging behaviour, improved level of functioning and quality of life based on the model.

A study[14] showed that positive behavioural outcomes may be achieved by an embedded IST model in which a core team of community intellectual disability service (CIDS) staff trained in managing challenging behaviour meet together regularly to discuss referrals with specialist supervision and peer support by the lead clinician. Literature from other population groups (e.g. dementia care)[15] and adult mental health[16] suggests that home treatment teams may be effective in managing crises and reducing admissions. Wheeler et al[17] showed that stakeholders in adult mental healthcare have a number of expectations from crisis resolution teams and this is likely to be the case for ISTs in the field of ID. So far, there has been limited reporting on stakeholder experiences of ISTs [14,17], so little is known whether service users and paid and family carers find the involvement of IST staff and frequency of contact helpful and acceptable.

As part of the Transforming Care programme[18], NHS England has prioritised ISTs as a central pillar in the arsenal of support for individuals who display challenging behaviour, and has invested in a variety of formats of such teams in areas with high admission rates. The study described here is the phase 2 of a wider project examining the implementation of ISTs in England. Stage 1 identified two IST models[19]: enhanced provision (model 1) and independent provision (model 2). The components of each model are shown below (table 1).

Table 1: Characteristics of IST models

**Intensive Support Teams (ISTs)**	
Model 1 – enhanced provision	Model 2 – independent provision
Integrated within the broader Community Intellectual Disability Services	Separate from Community Intellectual Disability Services
Long-term support (more than 6 months)	Short-term support (3-12 months)
Accept self-referrals	Referral via professionals
Large case-load (20+)	Small caseload (up to 15)
Less likely to use outcome measures	Use of outcome measures

Thus far, there is limited evidence for the comparative clinical and cost effectiveness of either enhanced or independent provision[14]. Evidence about long term outcomes is also lacking, as the length of follow up reported in published literature is limited (3-6 months), and therefore, it is possible that gains made during engagement with the IST are not maintained after discharge or after transfer to other services. Furthermore, patients and their carers may

For peer review only - http://bmjopen.bmj.com/site/about/guidelines.xhtml

face disruption and discontinuity in care due to frequent changes in service provision and may be dissatisfied with what they perceive as less “expert” service provided by CIDS teams [20].

Objectives

The overall aim of the study is to investigate patient outcomes (e.g. challenging behaviour, risk, adaptive behaviour, service use, quality of life) and service level outcomes (e.g. engagement with the IST, satisfaction with referral process, number of referrals, experience of care provided).

Specific objectives

- To generate evidence regarding which IST model best supports improved outcomes for adults with behaviour that challenges.
- To estimate the costs of the two IST models and examine cost effectiveness.
- To explore views and experiences of ISTs among relevant stakeholders (service users, paid and family carers, and service providers).
- To investigate how ISTs impact the lives of adults with intellectual disabilities who display challenging behaviour, their families and the local services.

Findings will generate evidence to inform and support decision making on commissioning of ISTs for adults with intellectual disability who display challenging behaviour.

Methods and analysis

We will compare the two models, enhanced provision and independent provision, respectively, of ISTs already identified in Phase 1 of the study[19]. We will collect study participant level outcomes at two assessment points (baseline and 9 months) and service level outcomes over the same time period. We will also collect qualitative data to understand the experiences and views of key stakeholders, and stakeholder experiences of the different models.

Setting and participants

Eight enhanced service provision ISTs and eight independent service provision ISTs (table 1) will be selected at random from ISTs surveyed in Phase 1 of the study[19]. Service users either already under the care of the 16 ISTs or newly referred and their caregivers, will be identified by IST staff either at first clinical assessment or from the IST services caseloads and asked if they are interested in taking part in the study. Following an expression of interest, the researchers will contact them to provide information about the study and to obtain written or audio recorded informed consent from those agreeing to undergo further research assessments.

For peer review only - http://bmjopen.bmj.com/site/about/guidelines.xhtml

Inclusion Criteria

a) Service: IST adheres to one of the identified models (table 1); has been operational for at least 12 months; there is commitment to fund it for the study duration; and it can achieve the sample size estimates.
b) Service users: Receive support from IST service; mild to profound intellectual disability based on clinical diagnosis; aged 18 years and over.

Exclusion Criteria

a) Service: Has been operational for less than 12 months at selection or there are plans to dissolve it.
b) Service users: Primary clinical diagnosis of personality disorder or substance misuse; decision by clinical team a referral to the study would be inappropriate (e.g. due to ongoing legal challenges to the service or the person being acutely unwell).

Consent

Consent will be sought from service users and their respective carers (paid and family) and/or nominated consultees. For those service users with decision making capacity, the researcher will speak to the potential participant by phone or in person and will give or send the service user participant information sheet (in Easy-Read format) to inform them of the study. If the service user agrees to take part in the study the individual will complete a written consent form or consent will be taken by telephone and audio recorded for purposes of verifying consent. The researcher will then repeat the above process (using the Carer participant information sheet) with the service users' paid or family carer to seek their consent to take part and complete the rest of the study measures. For those service users lacking decision making capacity, the researcher will approach the personal or nominated consultee for that person (using the Consultee information sheet) and seek written or audio recorded advice about including the service user in the study.

Outcome measures

Study participants level outcomes

Each participant will be recruited at any point of IST care and will be assessed at baseline and at 9 months which reflects the time period an IST may be expected to be involved in working with a participant. This length of follow up was chosen because our research and clinical experience indicates that, whilst there is a range of duration for IST involvement, assessment and delivery of behaviour support plans usually require about 3 months to

For peer review only - http://bmjopen.bmj.com/site/about/guidelines.xhtml

complete and adults with intellectual disabilities and challenging behaviour often remain in the care of the IST for approximately 12 months.

The primary outcome is challenging behaviour measured by the Aberrant Behaviour Checklist-Community (ABC-C)[21] which has been widely used in the target population and is completed by the caregiver (e.g., family, paid care home staff, IST staff).

A range of secondary endpoints examine the following: The carer reported Psychopathology Assessment for Adults with Developmental Disabilities checklist (PASADD Checklist)[22] is a mental health screening tool which provides sufficient information on potential mental health comorbidity that is often under-ascertained in adults with intellectual disability. The Threshold Assessment Grid (TAG)[23] measures clinical risk in adults with ID. The Quality of Life (QoL)[24] will be used with the individual with intellectual disability and/or the caregiver, if participants lack capacity, to measure the individual's quality of life. The Adaptive Behaviour Scale – Short Form assess level of functioning[25]. The Client Service Receipt Inventory (CSRI)[26] (adapted for the study, six month retrospective service use at each assessment point) is a widely used service use questionnaire and has been validated for use in mental health and intellectual disability services research.

Health related quality of life will be measured by the EQ-5D-5L (5 level)[27]. The EQ-5D is used to generate quality adjusted life years (QALYs). The self-report version will be used with the individual with intellectual disability where possible, and the proxy version will be used with the carergiver in the cases of participants lacking capacity.

We will collect sociodemographic information, number of hospital admissions and maintenance of accommodation at follow up will be recorded during the study period.

The study flow chart is shown in figure 1.

Service level outcomes

We will collect data from all participating ISTs including the number of people referred; proportion who engage with IST; time to 1st assessment and delivery of management plan; other IST scope (e.g. days of training given) and other engagement with local services (e.g. joint assessments with crisis teams) that will provide additional information about the ISTs caseload size. We shall map our service data onto the reports from the Mental Health and Learning Disabilities Data Set (MHLDDS) over the study period which provides information on hospital admissions and compared with admissions by IST model. This will provide a proxy measure of IST model impact on admissions.

For peer review only - http://bmjopen.bmj.com/site/about/guidelines.xhtml

Referrer's questionnaire

Practitioners from community intellectual disability services that frequently refer to ISTs across all sites will be invited to complete a brief questionnaire designed to capture satisfaction with referral processes, reasons for referrals, and number of referrals to IST. The questionnaire will be administered either online, via e-mail or post or a combination of these.

Qualitative sub-study: Stakeholder's views of ISTs

An embedded qualitative sub-study will allow us to investigate how IST care is experienced by service users and family carers, and to obtain a multi-perspective view of IST functioning within local service contexts, based on user, carer and practitioner views. Stakeholders include: i) IST managers and staff, ii) service users, and iii) family and paid carers. Data will be collected using semi-structured interviews with managers, staff, service users and carers. In order to link stakeholder perspectives to specific service contexts, the majority of data collection will be conducted in 7 IST "case study" sites. These sites will be selected from the larger pool of the 16 services, with an aim to include services that vary in terms of team size, caseload size and styles of working. Four services will be selected as case studies of "independent" ISTs and three services as case studies of "enhanced model" ISTs.

Across all stakeholder groups, interview schedules will be designed to explore views and experiences of the role and functioning of ISTs, and how they interface with other health and social care provision within their local context. Questions will cover the benefits, limitations and functioning of ISTs, and explore the factors that might affect these. We aim to conduct semi-structured interviews with up to 8 service users per IST model, including those who may need support to communicate (provided by family or paid carers); and up to 8 carers (a mixture of family and paid) per IST model. We will also attempt to interview a small number of family carers who have recently declined IST contact. Where possible, purposive sampling will be employed, in order to include a range of ages, work experience, professional backgrounds, and carer roles and relationships in our sample. In order to gain a broad perspective on IST implementation in England, we will aim to conduct interviews with all managers of the IST services involved in the main study. Interviews with IST managers and staff will be conducted in the early stages of the research study; interviews with service users and family and paid carers will be conducted near the time of discharge from IST services or around the 9 month follow up, whichever is sooner.

Study termination

The end of the study will be the date of the 9 months follow up assessment with the study participant. For those who take part in interviews, these will be arranged to take place shortly

For peer review only - http://bmjopen.bmj.com/site/about/guidelines.xhtml

after the questionnaire completion. Further, if the study failed to recruit adequately, it would be discussed with the Study Steering Committee and the funders as to whether it should stop or be extended.

Sample size

Based on two IST models, it was estimated that a sample of 96 participants per model (192 in total) would be required to detect a difference of 0.45 standard deviations (SD) in primary outcome score between the two models at the 5% significance level with 80% power and assuming an intraclass correlation (ICC) of 0.02[28]. After factoring in 15% loss to follow up, the estimated sample size required increased to 113 participants per model (226 participants in total).

Statistical analysis plan

The baseline characteristics of participants will be summarised separately for the two IST models. Categorical variables will be reported as counts and percentages, while continuous variables will be summarised as means and standard deviations (SD) or medians and interquartile ranges as appropriate depending on the distribution of the data.

The primary outcome is challenging behaviour measured by total score on the ABC. Change in ABC total score from baseline to 9 month follow up will be calculated for each participant. The mean difference in ABC score between the two IST models will be estimated from a multilevel linear regression model with change in ABC score as the outcome, a fixed effect of model as the main exposure, and a random effect of IST to account for clustering within services.

Age, gender, living arrangements, level of intellectual disability, level of risk, presence of autism and clinical comorbidities have been identified as potential confounders and will be specified as covariates in adjusted multilevel linear regression models similar to the main unadjusted model described above. The estimated difference in change in ABC total score from both unadjusted and adjusted models will be reported with accompanying 95% confidence interval (CI) and p-value.

The secondary outcomes detailed above will be analysed using statistical models analogous to those for the primary outcome. Binary outcomes will be analysed using multilevel logistic regression models. Non normally distributed continuous outcomes will be analysed using the bootstrap method and bias corrected 95% CI will be reported. P-values will not be reported for secondary outcomes.

For peer review only

Economic evaluation plan

We shall derive and report the costs of each IST service model over 9 months. To calculate the cost of each IST service model, we will use information on the description, time inputs of staff and related caseloads of professionals in the IST.

The cost effectiveness analyses will be conducted from a health and social care perspective and a wider societal perspective. Data on health and social care services and support provided by statutory services external to the ISTs will be obtained from the CSRI covering a retrospective period of 6 months. To service use and support data we shall attach unit costs taken from a wide range of sources[29-30]. Costs of unpaid care will be estimated from information on volume and type of support, the opportunity cost of lost work (wage rate) for carers in paid employment, and replacement cost for those not in paid employment based on cost of a home care worker. We shall extrapolate the six-month costs over the nine months period and examine the link between costs and sociodemographic, clinical characteristics of individuals in the study.

The cost-effectiveness of one IST model over another will be compared by calculating incremental cost-effectiveness ratios (ICERs), defined as difference in mean costs divided by difference in mean effects. Cost-effectiveness acceptability curves (CEAC)[31] will be plotted for each cost-outcome combination to show the likelihood of one IST model as cost-effective relative to another for a range of (implicit) values placed on incremental outcome improvements. Using the net benefit approach, monetary values of incremental effects and incremental costs are combined, and net benefit (NB) derived as: $NB = \lambda \times (effect_b - effect_a) - (cost_b - cost_a)$. Where, λ is the willingness-to-pay for a unit improvement in effectiveness (ABC, QALYs and QOL) and subscript 'a' and 'b' denote *IST model a* and *IST model b*, respectively. This approach allows costs and outcomes to be considered on the same monetary scale, taking account of sampling uncertainty and adjusting for baseline covariates and clustering.

For peer review only - http://bmjopen.bmj.com/site/about/guidelines.xhtml

Qualitative analysis plan

Interviews will be audio recorded and transcribed verbatim by an external agency. Data will be analysed using thematic analysis[32] conducted using NVivo software. A staged, collaborative, and primarily inductive analytic approach will be adopted, allowing us to iteratively develop a set of themes to capture key concerns and topics, as well as more abstract or underlying issues. Although numbers in stakeholder groups linked to each IST model may be relatively small, triangulation of the various stakeholder perspectives will allow us to obtain a broad picture of each IST model. Thus, we will be able to compare the IST models in terms of multiple stakeholders' views, as well as analysing the dataset as a whole to understand broadly common views and experiences of ISTs. Analysis will involve close collaboration between study researchers, the qualitative lead and other key members of the study team. The service user and carer group will also be consulted to provide their views on emerging themes and findings.

Patient and Public Involvement

A service-user reference group (Camden Disability Action) has supported the project and assisted with the research governance requirements including input to easy read versions of study materials (i.e., information sheets, consent forms) and piloting of instruments and qualitative interview topic guides with reference to people with intellectual disability. Family carer representatives from The Challenging Behaviour Foundation, an independent national charity of family carers of people with intellectual disabilities and challenging behaviour, have also been involved in the conduct of the study and advising on recruitment as well as reviewing study related information including the qualitative interviews topic guides. Meetings with both expert by experience groups are scheduled to take place every three months. Finally, there is service user and family caregiver representation at the Study Steering Committee.

Ethics

Ethical approval for this study was granted on 26 June 2018 by the London Bromley REC (18/LO/0890). All personal data are handled in accordance with the Data Protection Act. All outcome measures are stored electronically, and access is restricted to the principal investigator of the study and nominated researchers. All identifiable documents are saved as password protected files on password protected computers. Confidential information (including signed consent forms) are stored separately from paper records (e.g. Clinical Research Forms – CRFs) in locked filing cabinets in a restricted area accessed by members

For peer review only

For peer review only - http://bmjopen.bmj.com/site/about/guidelines.xhtml

of the UCL Division of Psychiatry. Data management and data quality checks will be performed by the research study team.

Dissemination plan

Our dissemination plan was developed in close collaboration with PPI representatives. Our dissemination and impact activities will include open-access academic publications in high-impact journals, presentations at academic and non-academic conferences, hosting a free public webinar about the study and producing briefings for carer groups, charities, and NHS services. A newsletter about the study progress will be sent to participants every 6 months and also available on the study website (https://www.ucl.ac.uk/psychiatry/research/epidemiology/pis/hassiotis-research-portfolio/intensive-support-teams). The research team will submit manuscripts to peer-reviewed publications, and authorship inclusion and order will be guided by levels of contribution. All publication material will acknowledge the funding contribution from NIHR. Requests for access to the anonymised data and statistical code should be addressed to the corresponding author once the study funder report and main papers are published.

Study progress

As has been mentioned the stage 1 of the project has been completed and reported[19]. The study has faced recruitment challenges which in the main included: reduced capacity to identify sufficient numbers of eligible participants due to small and static caseloads, services withdrawing or changing their remit halfway through the study, inadequate communication between ISTs and Clinical Research Networks which hampered research support. The number of ISTs was expanded to 23, 21 of which contributed in the study. The originally estimated 9 months of recruitment were extended to 17 with the last participant enrolled in May 2020; 130 9 moths follow ups have been completed. The last 4 months of recruitment occurred during the coronavirus pandemic and the subsequent lockdown which necessitated changes in study procedures including the introduction of remote consent and assessment processes. Thirteen of participants have been lost to follow up with one death from covid-19.

Authors' contributions

AH conceived the study, wrote the funding application and led the study design along with NM, RJ, KC, VC, IH, RR, LT, PL, VK, BLE who were the funding co-applicants. RJ developed the statistical element of the study. RR developed the health economic element of

For peer review only - http://bmjopen.bmj.com/site/about/guidelines.xhtml

the study. NM developed the qualitative analysis section of the study. VR drafted the first version of the protocol manuscript for publication with input from AH and RR. NK finalised the protocol paper and liaised with all authors. All authors approved the final protocol.

Funding

The study is funded by the National Institute of Health Research (NIHR) Health Services and Delivery Research (HS&DH) programme (project reference: 16/01/24) and is sponsored by University College London.

Competing interests

The authors declare no competing interests.

For peer review only

References

1 Maulik PK, Mascarenhas MN, Mathers CD, et al. Prevalence of intellectual disability: a meta-analysis of population-based studies. *Research in developmental disabilities* 2011;32:419-36.
2 Lowe K, Allen D, Jones E, et al. Challenging behaviours: Prevalence and topographies. *Journal of Intellectual Disability Research* 2007;51:625-36.
3 NHS England. *Building the right support: a national plan to develop community services and close inpatient facilities for people with a learning disability and/or autism who display behaviour that challenges, including those with a mental health condition*. NHS England 2015. https://www.england.nhs.uk/wp-content/uploads/2015/10/ld-nat-imp-plan-oct15.pdf (accessed 27 August 2019).
4 Cooper SA, Smiley E, Jackson A, et al. Adults with intellectual disabilities: prevalence, incidence and remission of aggressive behaviour and related factors. *Journal of intellectual disability research* 2009;53:217-32.
5 Davidson S, McGill P, Baker P, et al. A national UK survey of peripatetic support teams for children and adults with intellectual and developmental disability who display challenging behaviour. *International Journal of Positive Behaviour Support* 2015;5:26–33.
6 Hassiotis A. Community mental health services for individuals with intellectual disabilities. *Disease Management & Health Outcomes* 2002;10:409-17.
7 Department of Health. *The Mental Capacity Act.* Department of Health, 2005.
8 Lowe K, Felce D, Blackman D. People with learning disabilities and challenging behaviour: the characteristics of those referred and not referred to specialist teams. *Psychological Medicine* 1995;25:595-603.
9 Lowe K, Felce D, Blackman D. Challenging behaviour: the effectiveness of specialist support teams. *Journal of Intellectual Disability Research* 1996;40:336-47.
10 Hudson A, Wilken P, Jauernig R, et al. Regionally based teams for the treatment of challenging behaviour: A three-year outcome study. *Behaviour Change* 1995;12:209-15.
11 Hassiotis A, Robotham D, Canagasabey A, et al. Randomized, single-blind, controlled trial of a specialist behavior therapy team for challenging behavior in adults with intellectual disabilities. *American Journal of Psychiatry* 2009;166:1278-85.
12 Martin G, Costello H, Leese M, et al. An exploratory study of assertive community treatment for people with intellectual disability and psychiatric disorders: Conceptual,

For peer review only - http://bmjopen.bmj.com/site/about/guidelines.xhtml

clinical, and service issues. *Journal of Intellectual Disability Research* 2005;49:516–524.
13 Coelho RJ, Kelley PS, Deatsman-Kelly C. An experimental investigation of an innovative community treatment model for persons with a dual diagnosis (DD/MI). *The Journal of Rehabilitation* 1993;59:37.
14 Inchley-Mort S, Rantell K, Wahlich C, et al. Complex Behaviour Service: Enhanced model for challenging behaviour. *Advances in Mental Health and Intellectual Disabilities* 2014;8:219–227.
15 Toot S, Devine M, Orrell M. The effectiveness of crisis resolution/home treatment teams for older people with mental health problems: a systematic review and scoping exercise. *International journal of geriatric psychiatry* 2011;26:1221-30.
16 Murphy SM, Irving CB, Adams CE, Waqar M. Crisis intervention for people with severe mental illnesses. Cochrane Database of Systematic Reviews. 2015(12).
17 Wheeler C, Lloyd-Evans B, Churchard A, et al. Implementation of the Crisis Resolution Team model in adult mental health settings: a systematic review. *BMC psychiatry* 2015;15:74.
18 NHS England. *Transforming Care: Model Service Specifications: Supporting implementation of the service model* NHS England 2017 (https://www.england.nhs.uk/wp-content/uploads/2017/02/model-service-spec-2017.pdf). (accessed 25 March 2020).
19 Hassiotis A, Walsh A, Budgett J, et al. Intensive support for adults with intellectual disability and behaviours that challenge: a survey of provision and service typologies in England. *BJPsych Open* 2020;6.
20 Robotham D, King M, Canagasabey A, et al. Social validity of randomised controlled trials in health services research and intellectual disabilities: a qualitative exploration of stakeholder views. *Trials* 2011;12:144.
21 Aman MG. Aberrant Behavior Checklist: current identity and future developments. *Clin Exp Pharmacol* 2012;2(3):e114.
22 Prosser H, Moss S, Costello H, et al. Reliability and validity of the Mini PAS-ADD for assessing psychiatric disorders in adults with intellectual disability. *Journal of Intellectual Disability Research* 1998;42:264-72.
23 Slade M, Powell R, Rosen A, et al. Threshold Assessment Grid (TAG): the development of a valid and brief scale to assess the severity of mental illness. *Social psychiatry and psychiatric epidemiology* 2000;35:78-85.
24 Schalock RL, Keith, KD. Quality of Life Questionnaire Manual. Worthington, OH: IDS Publishing Corporation. 1993.
25 Hatton C, Emerson E, Robertson J, et al. The adaptive behavior scale-residential and community (part I): towards the development of a short form. *Research in Developmental Disabilities* 2001;22:273-88.
26 Beecham J. Collecting and estimating costs. *The Economic Evaluation of Mental Health Care Aldershot: Arena* 1995:157-174.
27 Herdman M, Gudex C, Lloyd A, et al. Development and preliminary testing of the new five-level version of EQ-5D (EQ-5D-5L). *Quality of life research*. 2011;20:1727-36.
28 Hassiotis A, Poppe M, Strydom A, et al. Clinical outcomes of staff training in positive behaviour support to reduce challenging behaviour in adults with intellectual disability: cluster randomised controlled trial. *The British Journal of Psychiatry*. 2018 Mar;212(3):161-8.
29 Department of Health. *NHS reference costs 2017-2018*, Department of Health, 2018.
30 Curtis L, Burns A. Unit costs of health and social care 2017, personal social services research unit, University of Kent, Canterbury, 2017.
31 Van Hout BA, Al MJ, Gordon GS, et al. Costs, effects and C/E-ratios alongside a clinical trial. *Health economics* 1994;3:309-19.
32 Braun V, Clarke V. Using thematic analysis in psychology. *Qualitative research in psychology* 2006;3(2):77-101.

For peer review only - http://bmjopen.bmj.com/site/about/guidelines.xhtml

Figure 1 Study flow chart

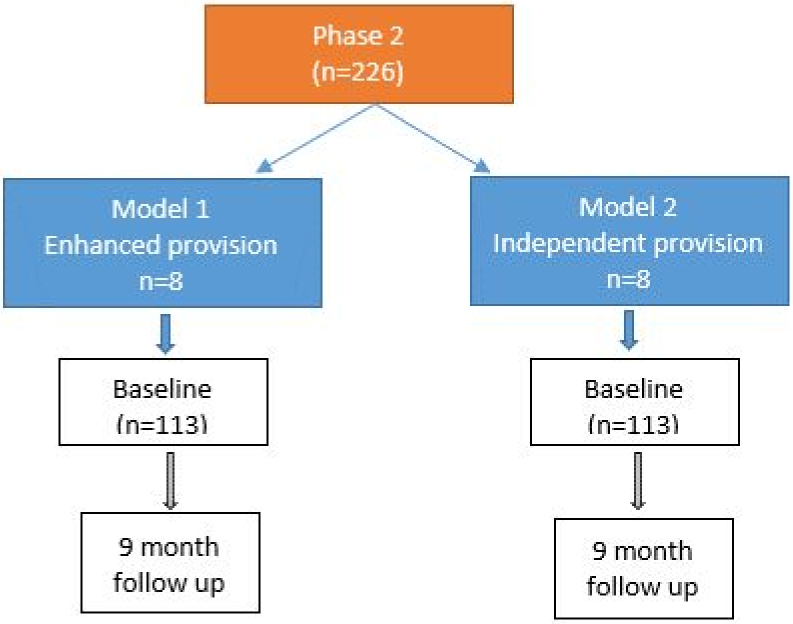

review only

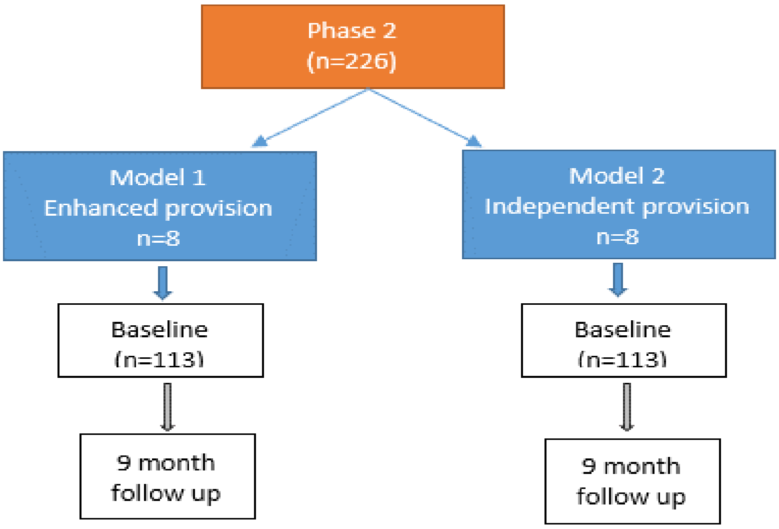

peer review only

BMJ Open

Clinical and cost evaluation of intensive support team (IST) models for adults with intellectual disabilities who display challenging behaviour: a comparative cohort study protocol

Journal:	*BMJ Open*
Manuscript ID	bmjopen-2020-043358.R1
Article Type:	Protocol
Date Submitted by the Author:	29-Dec-2020

Complete List of Authors:	HASSIOTIS, ANGELA; University College London, Division of Psychiatry<br>Kouroupa, Nancy; University College London, Division of Psychiatry<br>Jones, Rebecca; University College London, Division of Psychiatry<br>Morant, Nicola; University College London, Division of Psychiatry<br>Courtenay, Ken; Barnet Enfield and Haringey Mental Health NHS Trust<br>Hall, Ian; East London NHS Foundation Trust<br>Crossey, Vicky; NHS Lothian<br>Romeo, Renee; King's College London, Institute of Psychiatry Psychology and Neuroscience<br>Taggart, Laurence; University of Ulster Institute of Nursing and Health Research<br>Langdon, Peter; University of Warwick Centre for Educational Development Appraisal and Research<br>Ratti, Victoria; University College London, Division of Psychiatry<br>Kirchner, Vincent; Camden and Islington NHS Foundation Trust<br>Lloyd-Evans, Brynmor ; UCL, Division of Psychiatry

**Primary Subject Heading**:	Mental health
Secondary Subject Heading:	Mental health, Medical management
Keywords:	PSYCHIATRY, Adult psychiatry < PSYCHIATRY, Delirium & cognitive disorders < PSYCHIATRY, Organisation of health services < HEALTH SERVICES ADMINISTRATION & MANAGEMENT

SCHOLARONE™ Manuscripts

I, the Submitting Author has the right to grant and does grant on behalf of all authors of the Work (as defined in the below author licence), an exclusive licence and/or a non-exclusive licence for contributions from authors who are: i) UK Crown employees; ii) where BMJ has agreed a CC-BY licence shall apply, and/or iii) in accordance with the terms applicable for US Federal Government officers or employees acting as part of their official duties; on a worldwide, perpetual, irrevocable, royalty-free basis to BMJ Publishing Group Ltd ("BMJ") its licensees and where the relevant Journal is co-owned by BMJ to the co-owners of the Journal, to publish the Work in this journal and any other BMJ products and to exploit all rights, as set out in our licence.

The Submitting Author accepts and understands that any supply made under these terms is made by BMJ to the Submitting Author unless you are acting as an employee on behalf of your employer or a postgraduate student of an affiliated institution which is paying any applicable article publishing charge ("APC") for Open Access articles. Where the Submitting Author wishes to make the Work available on an Open Access basis (and intends to pay the relevant APC), the terms of reuse of such Open Access shall be governed by a Creative Commons licence – details of these licences and which Creative Commons licence will apply to this Work are set out in our licence referred to above.

Other than as permitted in any relevant BMJ Author's Self Archiving Policies, I confirm this Work has not been accepted for publication elsewhere, is not being considered for publication elsewhere and does not duplicate material already published. I confirm all authors consent to publication of this Work and authorise the granting of this licence.

For peer review only

Clinical and cost evaluation of intensive support team (IST) models for adults with intellectual disabilities who display challenging behaviour: a comparative cohort study protocol

Angela Hassiotis[1], Nancy Kouroupa[1], Rebecca Jones[1], Nicola Morant[1], Ken Courtenay[2], Ian Hall[4], Elisabeth Victoria Crossey[3], Renee Romeo[5], Laurence George Taggart[6], Peter E. Langdon[7], Victoria Ratti[1], Vincent Kirchner[8] and Brynmor Lloyd-Evans[1]

Author affiliations

1. Division of Psychiatry, University College London, UK
2. Barnet, Enfield and Haringey Mental Health NHS Trust, London, UK
3. NHS Lothian, UK
4. East London NHS Foundation Trust, UK
5. Institute of Psychiatry Psychology and Neuroscience, King's College London, UK
6. Institute of Nursing and Health Research, Ulster University, UK
7. Centre for Educational Development, Appraisal and Research, University of Warwick, UK and Coventry and Warwickshire Partnership NHS Trust
8. Camden and Islington NHS Foundation Trust, UK

Correspondence to

Nancy Kouroupa, UCL Division of Psychiatry, Maple House, 149 Tottenham Court Road, London, W1T 7NF, UK. Email: athanasia.kouroupa.12@ucl.ac.uk

For peer review only - http://bmjopen.bmj.com/site/about/guidelines.xhtml

Abstract

Introduction

Approximately 17% of adults with intellectual disabilities living in the community display behaviours that challenge. Intensive Support Teams (ISTs) have been recommended to provide high quality responsive care aimed at avoiding unnecessary admissions and reducing lengthy inpatient stays in England. We have identified two models of ISTs (model 1: enhanced provision and model 2: independent provision). This study aims to investigate the clinical and cost effectiveness of the two models of ISTs.

Methods and analysis

A cohort of 226 adults with intellectual disability (ID) displaying behaviour that challenges who receive support from ISTs from each model will be recruited and assessed at baseline and 9 months later to compare the clinical and cost effectiveness between models. The primary outcome is reduction in challenging behaviour measured by the Aberrant Behaviour Checklist-Community (ABC-C). The mean difference in change in ABC score between the two IST models will be estimated from a multilevel linear regression model. Secondary outcomes include mental health status, clinical risk, quality of life, health related quality of life, level of functioning and service use. We will undertake a cost effectiveness analysis taking both a health and social care and wider societal perspective. Semi-structured interviews will be conducted with multiple stakeholders (i.e., service users, paid/family carers, IST managers/staff) to investigate the experience of IST care as well as an online survey of referrers to capture their contact with the teams.

Ethics and dissemination

The study was approved by the London–Bromley Research Ethics committee (REC reference: 18/LO/0890). Informed consent will be obtained from the person with intellectual disability, or a family/nominated consultee for those lacking capacity and from his/her caregivers. The findings of the study will be disseminated to academic audiences, professionals, experts by

For peer review only

For peer review only - http://bmjopen.bmj.com/site/about/guidelines.xhtml

experience and arm's length bodies and policy makers via publications, seminars, and digital platforms.

Registration number

ClinicalTrials.gov Identifier: NCT03586375

Strengths and limitations of this study

- This study will be the first systematic national evaluation of IST models for adults with intellectual disability who display challenging behaviour.
- Accessing the experience of stakeholders (e.g. IST managers, staff members, family and paid carers and service users) will enable an in depth understanding of how ISTs function and how they respond to people with complex needs.
- This is not a randomised controlled trial so may be subject to biases associated with observational research.

Keywords

Intellectual disability, challenging behaviour, adult, intensive support, positive behaviour

For peer review only

For peer review only - http://bmjopen.bmj.com/site/about/guidelines.xhtml

Introduction

People with intellectual (also called learning) disabilities (ID), characterised by cognitive delay and difficulties in adaptive functioning, constitute approximately 1% of the population[1]. Seventeen percent of adults with intellectual disabilities, living in the community present with serious challenging behaviour including aggression, self-injury or other socially inappropriate behaviours[2]. As many as 100,000 children and adults are estimated to be at risk of admission to inpatient care due to the presence of such behaviours if they are not effectively supported in the community[3]. There are ongoing concerns that these individuals are subject to increased rates of hospitalisation, unnecessary long term use of psychotropic medication, poorer health, abuse and exclusion[4].

Intensive Support Teams (ISTs) are specialist teams which have been advocated for many years as the best services to help people with intellectual disabilities and challenging behaviour remain within their local communities. The teams may be staffed by one or more professions (e.g. psychology, nursing, psychiatry). They deliver interventions such as positive behaviour support, support adults with intellectual disabilities who are in a mental or behavioural crisis, and provide inreach when a person is admitted to a psychiatric inpatient facility[5-6]. ISTs are recommended to provide high quality proactive and responsive care aimed at avoiding unnecessary admissions or reducing inpatient length of stay and maintaining people in their local community[6-7]. However, there is little evidence to recommend a preferred IST model or way of implementing these aims, and there has not been a comprehensive attempt to describe the clinical and cost effectiveness of ISTs. Such information would be needed by health funders in order to fund services fit for purpose. The National Institute of Health and Care Excellence (NICE) Guideline (NG11)[8] reports that: *"It is widely recognised that locally accessible care settings could be beneficial and could reduce costs but there is no strong empirical evidence to support this"*.

Previous studies describe either demonstration projects following the closure of institutions[9-10] or region wide implementation of stand-alone services[11]. Three small controlled trials have examined respectively: 1) a stand-alone specialist support service delivering standard treatment in addition to behaviour therapy in one area in England[12]; 2) a stand-alone team delivering assertive outreach for adults with intellectual disabilities who display challenging behaviour in inner London[13]; and 3) an active case management model[14]. Comparator treatment was usual care in all three studies. The remit of the ISTs differed between studies, with one providing support to people who display challenging behaviour whilst the other two provided a replication of the intensive support management from adult mental health care. These small studies presented equivocal findings in terms of

For peer review only - http://bmjopen.bmj.com/site/about/guidelines.xhtml
For peer review only

reduction in challenging behaviour, improved level of functioning and quality of life dependent on the model.

A study[15] showed that positive behavioural outcomes may be achieved by an embedded IST model in which a core team of community intellectual disability service (CIDS) staff trained in managing challenging behaviour meet together regularly to discuss referrals with specialist supervision and peer support by the lead clinician. Literature from other population groups (e.g. dementia care)[16] and adult mental health[17] suggests that home treatment teams may be effective in managing crises and reducing admissions. Wheeler et al[18] showed that stakeholders in adult mental healthcare have a number of expectations from crisis resolution teams and this is likely to be the case for ISTs in the field of ID. So far, there has been limited reporting on stakeholder experiences of ISTs [15,18], so little is known whether service users and paid and family carers find the involvement of IST staff and frequency of contact helpful and acceptable.

As part of the Transforming Care programme[19], NHS England has prioritised ISTs as a central pillar in the arsenal of support for individuals who display challenging behaviour, and has invested in a variety of formats of such teams in areas with high admission rates. The current study is the second phase of a wider project that set out to systematically examine the implementation of ISTs in England.

The first phase identified two IST service models[20]: enhanced provision (model 1) and independent provision (model 2) following an England-wide survey of ISTs (n=73)[20]. The components of each model as reported by professionals in the survey are shown below (table 1).

Table 1: Characteristics of IST models

Intensive Support Teams (ISTs)	
**Model 1 – enhanced provision**	**Model 2 – independent provision**
Integrated within the broader Community Intellectual Disability Services	Separate from Community Intellectual Disability Services
Long-term support (more than 6 months)	Short-term support (3-12 months)
Accept self-referrals	Referral via professionals
Large case-load (20+)	Small caseload (up to 15)
Less likely to use outcome measures	Use of outcome measures

For peer review only - http://bmjopen.bmj.com/site/about/guidelines.xhtml

There is limited evidence for the comparative clinical and cost effectiveness of either enhanced or independent provision[15] and of long-term clinical gains. Furthermore, satisfaction of stakeholders with the ISTs has hardly featured in the literature and that deserves better understanding if the ISTs are fit for purpose and responsive to patient need.

Objectives

The overall aim of phase 2 of the study is to investigate patient outcomes (e.g. challenging behaviour, risk, adaptive behaviour, service use, quality of life) and service level outcomes (e.g. engagement with the IST, satisfaction with referral process, number of referrals, experience of care provided).

Specific objectives

- To generate evidence regarding which IST model best supports improved outcomes for adults who display challenging behaviour
- To estimate the costs of the two IST models and examine cost effectiveness.
- To explore views and experiences of ISTs among relevant stakeholders (service users, paid and family carers, and service providers).
- To investigate how ISTs impact the lives of adults with intellectual disabilities who display challenging behaviour in their caseload, their families and the local services.

Findings will generate evidence to inform and support decision making on commissioning of ISTs for adults with intellectual disability who display challenging behaviour.

Methods and analysis

The research team will compare the two models, enhanced provision and independent provision, respectively, of ISTs already identified in Phase 1 of the study[20]. The research assistant or clinical study officers will collect study participant level outcomes at two assessment points (baseline and 9 months) and service level outcomes over the same time period. The research assistant will also collect qualitative data to understand the experiences and views of key stakeholders, and stakeholder experiences of the different models.

Setting and participants

Eight enhanced service provision ISTs and eight independent service provision ISTs (table 1) will be selected at random from ISTs surveyed in Phase 1 of the study[20]. Service users either already under the care of the 16 ISTs or newly referred and their caregivers, will be identified by IST staff either at first clinical assessment or from the IST services caseloads and asked if they are interested in taking part in the study. Following an expression of interest, the researchers will contact them to provide information about the study and to

For peer review only - http://bmjopen.bmj.com/site/about/guidelines.xhtml

obtain written or audio recorded informed consent from those agreeing to undergo further research assessments.

Inclusion Criteria

a) Service: IST adheres to one of the identified models (table 1); has been operational for at least 12 months; there is commitment to fund it for the study duration; and it can achieve the sample size estimates.
b) Service users: Receive support from IST service; mild to profound intellectual disability based on clinical diagnosis; aged 18 years and over.

Exclusion Criteria

a) Service: Has been operational for less than 12 months at selection or there are plans to dissolve it.
b) Service users: Primary clinical diagnosis of personality disorder or substance misuse; decision by clinical team a referral to the study would be inappropriate (e.g. due to ongoing legal challenges to the service or the person being acutely unwell).

Consent

Consent will be sought from service users and their respective carers (i.e., paid and family) and/or nominated consultees where the participant with intellectual disability lacks capacity in accordance with the Mental Capacity Act 2005[8]. For those service users with decision making capacity, the researcher will speak to the potential participant by phone or in person and will give or send the service user participant information sheet (in Easy-Read format) to inform them of the study. If the service user agrees to take part in the study the individual will complete a written consent form or consent will be taken by telephone and audio recorded for purposes of verifying consent. The researcher will then repeat the above process (using the Carer participant information sheet) with the service users' paid or family carer to seek their consent to take part and complete the rest of the study measures. For those service users lacking decision making capacity, the researcher will approach the personal or nominated consultee for that person (using the Consultee information sheet) and seek written or audio recorded advice about including the service user in the study.

Outcome measures

Study participants level outcomes

Each participant will be recruited at any point of IST care and will be assessed at baseline and at 9 months which reflects the time period an IST may be expected to be involved in working with a participant. This length of follow up was chosen because our research and

For peer review only - http://bmjopen.bmj.com/site/about/guidelines.xhtml

clinical experience indicates that, whilst there is a range of duration for IST involvement, assessment and delivery of behaviour support plans usually require about 3 months to complete and adults with intellectual disabilities and challenging behaviour often remain in the care of the IST for approximately 12 months.

The primary outcome is challenging behaviour measured by the Aberrant Behaviour Checklist-Community (ABC-C)[21] which has been widely used in the target population and is completed by the caregiver (e.g., family, paid care home staff, IST staff).

A range of secondary endpoints examine the following: The carer reported Psychopathology Assessment for Adults with Developmental Disabilities checklist (PASADD Checklist)[22] is a mental health screening tool which provides sufficient information on potential mental health comorbidity that is often under-ascertained in adults with intellectual disability. The Threshold Assessment Grid (TAG)[23] measures clinical risk in adults with ID. The Quality of Life (QoL)[24] will be used with the individual with intellectual disability and/or the caregiver, if participants lack capacity, to measure the individual's quality of life. The Adaptive Behaviour Scale – Short Form assesses level of functioning[25]. The Client Service Receipt Inventory (CSRI)[26] (adapted for the study, six month retrospective service use at each assessment point) is a widely used service use questionnaire and has been validated for use in mental health and intellectual disability services research.

Health related quality of life will be measured by the EQ-5D-5L (5 level)[27]. The EQ-5D is used to generate quality adjusted life years (QALYs). The self-report version will be used with the individual with intellectual disability where possible, and the proxy version will be used with the carergiver in the cases of participants lacking capacity.

The researchers will collect sociodemographic information, as well as number of hospital admissions and maintenance of accommodation at follow up.

The study participant flow chart is shown in figure 1.

Service level outcomes

The researchers will collect data from all participating ISTs including the number of people referred; proportion who engage with IST; time to 1st assessment and delivery of management plan; other IST scope (e.g. days of training given) and other engagement with local services (e.g. joint assessments with crisis teams) that will provide additional information about the ISTs caseload size. We will map our service data onto the reports from the Mental Health and Learning Disabilities Data Set (MHLDDS) over the study period which provides information on

For peer review only - http://bmjopen.bmj.com/site/about/guidelines.xhtml

hospital admissions and compares with admissions by IST model. This will provide a proxy measure of IST model impact on admissions.

Referrer's questionnaire

Practitioners from community intellectual disability services that frequently refer to ISTs across all sites will be invited to complete a brief questionnaire designed to capture satisfaction with referral processes, reasons for referrals, and number of referrals to IST. The questionnaire will be administered either online, via e-mail or post or a combination of these.

Qualitative sub-study: Stakeholder's views of ISTs

An embedded qualitative sub-study will allow to investigate how IST care is experienced by service users and family carers, and to obtain a multi-perspective view of IST functioning within local service contexts, based on user, carer and practitioner views. The researchers will conduct semi-structured interviews with i) IST managers and staff, ii) service users, and iii) family and paid carers. Up to eight service users per IST model, including those who may need support to communicate (provided by family or paid carers); and up to eight carers (a mixture of family and paid) per IST model will be included. The researchers will also attempt to interview a small number of family carers who have recently declined IST contact. Purposive sampling will be employed, in order to include a range of ages, work experience, professional backgrounds, and carer roles and relationships in our sample. In order to gain a broad perspective on IST implementation in England, the researchers will aim to conduct interviews with all managers of the IST services taking part in the phase 2. Interviews with IST managers and staff will be conducted in the early stages of the research study; interviews with service users and family and paid carers will be conducted near the time of discharge from IST services or around the 9 month follow up, whichever is sooner.

The interview will take place at a location convenient (i.e., home, clinic, or remotely with the aid of technology) for the participant and will be audio-recorded. Across all stakeholder groups, interview schedules will be designed to explore views and experiences of the role and functioning of ISTs, and how they interface with other health and social care provision within their local context. The semi-structured interview schedule will address the operational functioning, benefits, and limitations of ISTs, and explore the factors that might affect these following consultation with the patient and public involvement representatives (PPI). In order to link stakeholder perspectives to specific service contexts, we aim to carry out data collection within seven IST "case study" sites. These sites will be selected from the larger pool of the 16 services, with an aim to include services that vary in terms of team size, caseload size and styles of working based on the reflections and other relevant information available to the research team. Four services will be selected as case studies of

For peer review only - http://bmjopen.bmj.com/site/about/guidelines.xhtml

“independent” ISTs and three services as case studies of “enhanced model” ISTs. The use of case study sites is appropriate where the research focus is to assess “mega systems” of great complexity[28].

Study termination

The end of the study will be the date of the 9 months follow up assessment with the study participant. For those who take part in interviews, these will be arranged to take place shortly after the questionnaire completion. Further, if the study failed to recruit adequately, it would be discussed with the Study Steering Committee and the funders as to whether it should stop or be extended.

Sample size

Based on two IST models, it was estimated that a sample of 96 participants per model (192 in total) would be required to detect a difference of 0.45 standard deviations (SD) in primary outcome score between the two models at the 5% significance level with 80% power and assuming an intraclass correlation (ICC) of 0.02[12,29]. After factoring in 15% loss to follow up, the estimated sample size required increased to 113 participants per model (226 participants in total).

Statistical analysis plan

As per the pre-determined statistical analysis plan, the baseline characteristics of participants will be summarised separately for the two IST models. Categorical variables will be reported as counts and percentages, while continuous variables will be summarised as means and standard deviations (SD) or medians and interquartile ranges as appropriate depending on the distribution of the data.

The primary outcome is challenging behaviour measured by total score on the ABC. Change in ABC total score from baseline to 9 month follow up will be calculated for each participant. The mean difference in ABC score between the two IST models will be estimated from a multilevel linear regression model with change in ABC score as the outcome, a fixed effect of model as the main exposure, and a random effect of IST to account for clustering within services.

Age, gender, living arrangements, level of intellectual disability, level of risk, presence of autism and clinical comorbidities have been identified as potential confounders and will be specified as covariates in adjusted multilevel linear regression models similar to the main unadjusted model described above. The estimated difference in change in ABC total score from both unadjusted and adjusted models will be reported with accompanying 95% confidence interval (CI) and p-value.

For peer review only - http://bmjopen.bmj.com/site/about/guidelines.xhtml

The secondary outcomes detailed above will be analysed using statistical models analogous to those for the primary outcome. Binary outcomes will be analysed using multilevel logistic regression models. Non normally distributed continuous outcomes will be analysed using the bootstrap method and bias corrected 95% CI will be reported. P-values will not be reported for secondary outcomes.

Economic evaluation plan

As per the pre-determined health economic analysis plan, we shall derive and report the costs of each IST service model over 9 months. To calculate the cost of each IST service model, we will use information on the description, time inputs of staff and related caseloads of professionals in the IST.

The cost effectiveness analyses will be conducted from a health and social care perspective and a wider societal perspective. Data on health and social care services and support provided by statutory services external to the ISTs will be obtained from the CSRI covering a retrospective period of 6 months. To service use and support data we shall attach unit costs taken from a wide range of sources[30-31]. Costs of unpaid care will be estimated from information on volume and type of support, the opportunity cost of lost work (wage rate) for carers in paid employment, and replacement cost for those not in paid employment based on cost of a home care worker. We shall extrapolate the six-month costs over the nine months period and examine the link between costs and sociodemographic, clinical characteristics of individuals in the study.

The cost-effectiveness of one IST model over another will be compared by calculating incremental cost-effectiveness ratios (ICERs), defined as difference in mean costs divided by difference in mean effects. Cost-effectiveness acceptability curves (CEAC)[32] will be plotted for each cost-outcome combination to show the likelihood of one IST model as cost-effective relative to another for a range of (implicit) values placed on incremental outcome improvements. Using the net benefit approach, monetary values of incremental effects and incremental costs are combined, and net benefit (NB) derived as: NB = λ x (effectb - effecta) – (costb – costa). Where, λ is the willingness-to-pay for a unit improvement in effectiveness (ABC, QALYs and QOL) and subscript 'a' and 'b' denote *IST model a* and *IST model b*, respectively. This approach allows costs and outcomes to be considered on the same monetary scale, taking account of sampling uncertainty and adjusting for baseline covariates and clustering.

For peer review only - http://bmjopen.bmj.com/site/about/guidelines.xhtml

Qualitative analysis plan

Interviews will be audio recorded and transcribed verbatim by an external agency. Data will be analysed using thematic analysis[33] conducted using NVivo software. A staged, collaborative, and primarily inductive analytic approach will be adopted, allowing us to iteratively develop a set of themes to capture key concerns and topics, as well as more abstract or underlying issues. Although numbers in stakeholder groups linked to each IST model may be relatively small, triangulation of the various stakeholder perspectives will allow us to obtain a broad picture of each IST model. Thus, we will be able to compare the IST models in terms of multiple stakeholders' views, as well as analysing the dataset as a whole to understand broadly common views and experiences of ISTs. Analysis will involve close collaboration between study researchers, the qualitative lead and other key members of the study team. Service user and carer group meetings will be scheduled three times per year. The members of the advisory groups will receive easy read summary documents to be informed about the study progress, share views on different recruitment strategies, comment on the coding, analysis and interpretation of study findings.

Patient and Public Involvement

A service-user reference group (Camden Disability Action) has supported the project and assisted with the research governance requirements including input to easy read versions of study materials (i.e., information sheets, consent forms) and piloting of instruments and qualitative interview topic guides with reference to people with intellectual disability. Family carer representatives from The Challenging Behaviour Foundation, an independent national charity of family carers of people with intellectual disabilities and challenging behaviour, have also been involved in the conduct of the study and advising on recruitment as well as reviewing study related information including the qualitative interviews topic guides. Meetings with both expert by experience groups are scheduled to take place every three months. Finally, there is service user and family caregiver representation at the Study Steering Committee overseeing the study.

Ethics

Ethical approval for this study was granted on 26 June 2018 by the London Bromley REC (18/LO/0890). All personal data are handled in accordance with the Data Protection Act. All outcome measures are stored electronically, and access is restricted to the principal investigator of the study and nominated researchers. All identifiable documents are saved as password protected files on password protected computers. Confidential information (including signed consent forms) are stored separately from paper records (e.g. Clinical

For peer review only - http://bmjopen.bmj.com/site/about/guidelines.xhtml

Research Forms – CRFs) in locked filing cabinets in a restricted area accessed by members of the UCL Division of Psychiatry. Data management and data quality checks will be performed by the research study team.

Dissemination plan

Our dissemination plan was developed in close collaboration with PPI representatives. Our dissemination and impact activities will include open-access academic publications in high-impact journals, presentations at academic and non-academic conferences, hosting a free public webinar about the study and producing briefings for carer groups, charities, and NHS services. A newsletter about the study progress will be sent to participants every 6 months and also available on the study website (https://www.ucl.ac.uk/psychiatry/research/epidemiology/pis/hassiotis-research-portfolio/intensive-support-teams). The research team will submit manuscripts to peer-reviewed publications, and authorship inclusion and order will be guided by levels of contribution. All publication material will acknowledge the funding contribution from NIHR. Requests for access to the anonymised data and statistical code should be addressed to the corresponding author once the study funder report and main papers are published.

Study progress and covid-19 impact

The first phase of the study has been completed and reported[20]. The study has faced recruitment challenges which in the main included: reduced capacity to identify sufficient numbers of eligible participants due to small and static caseloads, services withdrawing or changing their remit halfway through the study, inadequate communication between ISTs and Clinical Research Networks which hampered research support. The number of ISTs was expanded to 23, 21 of which contributed participants to the study. The originally estimated 9 months of recruitment were extended to 17 with the last participant enrolled in May 2020; 180 9 month follow ups have been completed. The last 4 months of recruitment occurred during the coronavirus pandemic and the subsequent lockdown which necessitated changes in study procedures including the introduction of remote completion of the research assessments and qualitative interviews. Those changes were non-substantial amendments [Number 7] to the REC that had originally approved the study. Thirteen participants have been lost to follow up with one death from covid-19.

Authors' contributions

AH conceived the study, wrote the funding application and led the study design along with NM, RJ, KC, VC, IH, RR, LT, PL, VK, BLE who were the funding co-applicants. RJ

For peer review only - http://bmjopen.bmj.com/site/about/guidelines.xhtml

developed the statistical element of the study. RR developed the health economic element of the study. NM developed the qualitative analysis section of the study. VR drafted the first version of the protocol manuscript for publication with input from AH and RR. NK finalised the protocol paper and liaised with all authors. All authors approved the final protocol.

Funding

The study is funded by the National Institute of Health Research (NIHR) Health Services and Delivery Research (HS&DH) programme (project reference: 16/01/24). This publication presents independent research funded by the National Institute for Health Research (NIHR). The views expressed are those of the author(s) and not necessarily those of the NHS, the NIHR or the Department of Health and Social Care.

Competing interests

The authors declare no competing interests.

References

1 Maulik PK, Mascarenhas MN, Mathers CD, Dua T, Saxena S. Prevalence of intellectual disability: a meta-analysis of population-based studies. *Research in developmental disabilities*. 2011;32(2):419-36.
2 Lowe K, Allen D, Jones E, et al. Challenging behaviours: Prevalence and topographies. *Journal of Intellectual Disability Research* 2007;51:625-36.
3 NHS England. *Building the right support: a national plan to develop community services and close inpatient facilities for people with a learning disability and/or autism who display behaviour that challenges, including those with a mental health condition*. NHS England 2015. https://www.england.nhs.uk/wp-content/uploads/2015/10/ld-nat-imp-plan-oct15.pdf (accessed 27 August 2019).
4 Cooper SA, Smiley E, Jackson A, et al. Adults with intellectual disabilities: prevalence, incidence and remission of aggressive behaviour and related factors. *Journal of intellectual disability research* 2009;53:217-32.
5 Hassiotis A, Tyrer P, Oliver P. Psychiatric assertive outreach and learning disability services. Advances in Psychiatric Treatment. *Cambridge University Press*; 2003;9(5):368–73.
6 Hassiotis, A. Community mental health services for individuals with intellectual disabilities - issues and approaches to optimizing outcomes. *Disease Management and Health Outcomes*, 2002;10:409-417.
7 Davidson S, McGill P, Baker P, et al. A national UK survey of peripatetic support teams for children and adults with intellectual and developmental disability who display challenging behaviour. *International Journal of Positive Behaviour Support* 2015;5:26–33.
8 Department of Health. *The Mental Capacity Act.* Department of Health, 2005.
9 Lowe K, Felce D, Blackman D. People with learning disabilities and challenging behaviour: the characteristics of those referred and not referred to specialist teams. *Psychological Medicine* 1995;25:595-603.
10 Lowe K, Felce D, Blackman D. Challenging behaviour: the effectiveness of specialist support teams. *Journal of Intellectual Disability Research* 1996;40:336-47.
11 Hudson A, Wilken P, Jauernig R, et al. Regionally based teams for the treatment of

For peer review only - http://bmjopen.bmj.com/site/about/guidelines.xhtml

challenging behaviour: A three-year outcome study. *Behaviour Change* 1995;12:209-15.
12 Hassiotis A, Robotham D, Canagasabey A, et al. Randomized, single-blind, controlled trial of a specialist behavior therapy team for challenging behavior in adults with intellectual disabilities. *American Journal of Psychiatry* 2009;166:1278-85.
13 Martin G, Costello H, Leese M, et al. An exploratory study of assertive community treatment for people with intellectual disability and psychiatric disorders: Conceptual, clinical, and service issues. *Journal of Intellectual Disability Research* 2005;49:516–524.
14 Coelho RJ, Kelley PS, Deatsman-Kelly C. An experimental investigation of an innovative community treatment model for persons with a dual diagnosis (DD/MI). *The Journal of Rehabilitation* 1993;59:37.
15 Inchley-Mort S, Rantell K, Wahlich C, et al. Complex Behaviour Service: Enhanced model for challenging behaviour. *Advances in Mental Health and Intellectual Disabilities* 2014;8:219–227.
16 Toot S, Devine M, Orrell M. The effectiveness of crisis resolution/home treatment teams for older people with mental health problems: a systematic review and scoping exercise. *International journal of geriatric psychiatry* 2011;26:1221-30.
17 Murphy SM, Irving CB, Adams CE, Waqar M. Crisis intervention for people with severe mental illnesses. Cochrane Database of Systematic Reviews. 2015(12).
18 Wheeler C, Lloyd-Evans B, Churchard A, et al. Implementation of the Crisis Resolution Team model in adult mental health settings: a systematic review. *BMC psychiatry* 2015;15:74.
19 NHS England. *Transforming Care: Model Service Specifications: Supporting implementation of the service model* NHS England 2017 (https://www.england.nhs.uk/wp-content/uploads/2017/02/model-service-spec-2017.pdf). (accessed 25 March 2020).
20 Hassiotis A, Walsh A, Budgett J, et al. Intensive support for adults with intellectual disability and behaviours that challenge: a survey of provision and service typologies in England. *BJPsych Open* 2020;6.
21 Aman MG. Aberrant Behavior Checklist: current identity and future developments. *Clin Exp Pharmacol* 2012;2(3):e114.
22 Prosser H, Moss S, Costello H, et al. Reliability and validity of the Mini PAS-ADD for assessing psychiatric disorders in adults with intellectual disability. *Journal of Intellectual Disability Research* 1998;42:264-72.
23 Slade M, Powell R, Rosen A, et al. Threshold Assessment Grid (TAG): the development of a valid and brief scale to assess the severity of mental illness. *Social psychiatry and psychiatric epidemiology* 2000;35:78-85.
24 Schalock RL, Keith, KD. Quality of Life Questionnaire Manual. Worthington, OH: IDS Publishing Corporation. 1993.
25 Hatton C, Emerson E, Robertson J, et al. The adaptive behavior scale-residential and community (part I): towards the development of a short form. *Research in Developmental Disabilities* 2001;22:273-88.
26 Beecham J. Collecting and estimating costs. *The Economic Evaluation of Mental Health Care Aldershot: Arena* 1995:157-174.
27 Herdman M, Gudex C, Lloyd A, et al. Development and preliminary testing of the new five-level version of EQ-5D (EQ-5D-5L). *Quality of life research*. 2011;20:1727-36.
28 Yin RK. Enhancing the quality of case studies in health services research. Health services research. 1999;34:1209.
29 Hassiotis A, Poppe M, Strydom A, et al. Clinical outcomes of staff training in positive behaviour support to reduce challenging behaviour in adults with intellectual disability: cluster randomised controlled trial. *The British Journal of Psychiatry*. 2018;212:161-8.
30 Department of Health. *NHS reference costs 2017-2018*, Department of Health, 2018.
31 Curtis L, Burns A. Unit costs of health and social care 2017, personal social services research unit, University of Kent, Canterbury, 2017.

For peer review only - http://bmjopen.bmj.com/site/about/guidelines.xhtml

32 Van Hout BA, Al MJ, Gordon GS, et al. Costs, effects and C/E-ratios alongside a clinical trial. *Health economics* 1994;3:309-19.
33 Braun V, Clarke V. Using thematic analysis in psychology. *Qualitative research in psychology* 2006;3(2):77-101.

For peer review only

For peer review only - http://bmjopen.bmj.com/site/about/guidelines.xhtml

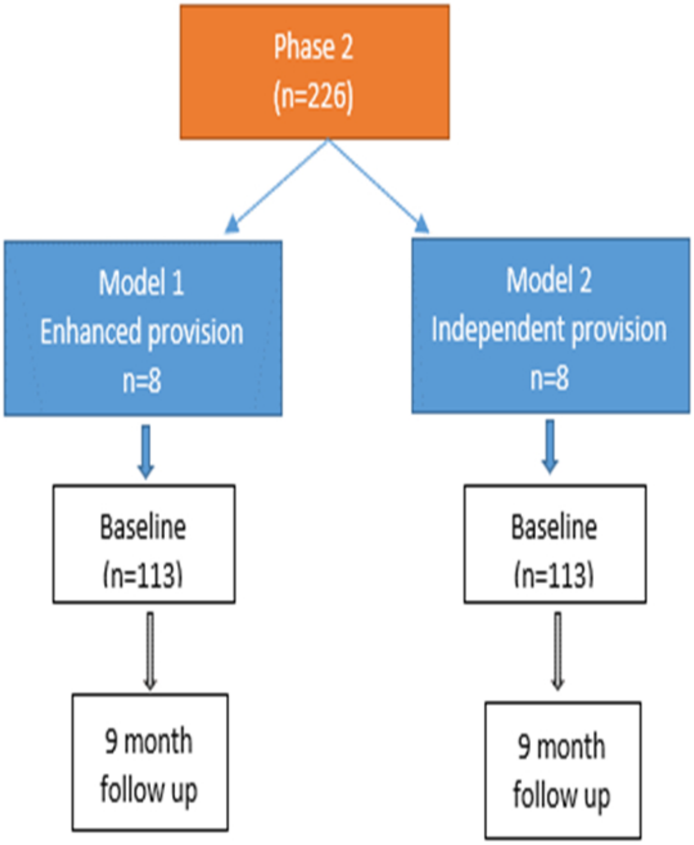

Study participant flow chart

89x89mm (220 x 220 DPI)
